# Comparison of the prognostic value of early-phase proton magnetic resonance spectroscopy and diffusion tensor imaging with serum neuron-specific enolase at 72 h in comatose survivors of out-of-hospital cardiac arrest—a substudy of the XeHypotheca trial

**DOI:** 10.1007/s00234-022-03063-z

**Published:** 2022-10-17

**Authors:** Kalle Koskensalo, Sami Virtanen, Jani Saunavaara, Riitta Parkkola, Ruut Laitio, Olli Arola, Marja Hynninen, Päivi Silvasti, Eija Nukarinen, Juha Martola, Heli M. Silvennoinen, Marjaana Tiainen, Risto O. Roine, Harry Scheinin, Antti Saraste, Mervyn Maze, Tero Vahlberg, Timo T. Laitio, for the XeHYPOTHECA Research Group

**Affiliations:** 1 Turku PET Centre, Turku University Hospital and University of Turku, Turku, Finland; 2 Department of Medical Physics, Turku University Hospital and University of Turku, Turku, Finland; 3 Department of Radiology, University of Turku, Turku University Hospital, Turku, Finland; 4 Division of Perioperative Services, Intensive Care Medicine and Pain Management, Turku University Hospital, University of Turku, POB 52, 20521 Turku, Finland; 5 Division of Intensive Care Medicine, Department of Anesthesiology, Intensive Care and Pain Medicine, University of Helsinki and Helsinki University Hospital, Helsinki, Finland; 6 Department of Radiology, University of Helsinki and Helsinki University Hospital, Helsinki, Finland; 7 Department of Neurology, University of Helsinki and Helsinki University Hospital, Helsinki, Finland; 8 Division of Clinical Neurosciences, University of Turku, Turku University Hospital, Turku, Finland; 9 Heart Centre, Turku University Hospital and University of Turku, Turku, Finland; 10 Department of Anesthesia and Perioperative Care, University of California, San Francisco, San Francisco, CA USA; 11 Department of Biostatistics, University of Turku and Turku University Hospital, Turku, Finland

**Keywords:** Diffusion tensor imaging, 1H-MRS, Neuron-specific enolase, Cardiac arrest, Brain hypoxia–ischemia, Prognostication

## Abstract

**Purpose:**

We compared the predictive accuracy of early-phase brain diffusion tensor imaging (DTI), proton magnetic resonance spectroscopy (1H-MRS), and serum neuron-specific enolase (NSE) against the motor score and epileptic seizures (ES) for poor neurological outcome after out-of-hospital cardiac arrest (OHCA).

**Methods:**

The predictive accuracy of DTI, 1H-MRS, and NSE along with motor score at 72 h and ES for the poor neurological outcome (modified Rankin Scale, mRS, 3 − 6) in 92 comatose OHCA patients at 6 months was assessed by area under the receiver operating characteristic curve (AUROC). Combined models of the variables were included as exploratory.

**Results:**

The predictive accuracy of fractional anisotropy (FA) of DTI (AUROC 0.73, 95% CI 0.62–0.84), total *N*-acetyl aspartate/total creatine (tNAA/tCr) of 1H-MRS (0.78 (0.68 − 0.88)), or NSE at 72 h (0.85 (0.76 − 0.93)) was not significantly better than motor score at 72 h (0.88 (95% CI 0.80–0.96)). The addition of FA and tNAA/tCr to a combination of NSE, motor score, and ES provided a small but statistically significant improvement in predictive accuracy (AUROC 0.92 (0.85–0.98) vs 0.98 (0.96–1.00), *p* = 0.037).

**Conclusion:**

None of the variables (FA, tNAA/tCr, ES, NSE at 72 h, and motor score at 72 h) differed significantly in predicting poor outcomes in this patient group. Early-phase quantitative neuroimaging provided a statistically significant improvement for the predictive value when combined with ES and motor score with or without NSE. However, in clinical practice, the additional value is small, and considering the costs and challenges of imaging in this patient group, early-phase DTI/MRS cannot be recommended for routine use.

**Trial registration:**

ClinicalTrials.gov NCT00879892, April 13, 2009.

**Supplementary Information:**

The online version contains supplementary material available at 10.1007/s00234-022-03063-z.

## Introduction

In-hospital mortality of successfully resuscitated out-of-hospital cardiac arrest (OHCA) patients remains high, ranging from 41 to 86%, despite the implementation of therapeutic hypothermia (also referred to as targeted temperature management) and other treatments [1, 2]. The major cause of morbidity and mortality in survivors of OHCA is hypoxic-ischemic brain damage with survivors at risk for a diverse spectrum of neurological injuries [3].

Based on the current guidelines, neurological prognostication is recommended in patients with a motor score ≤ 3 at 72 h or later after cardiac arrest. Notably, these guidelines allow imaging to be performed before 72 h from ROSC. However, the results of these early imaging studies should only be evaluated later, at the time of clinical determination [4]. A multimodal approach with a combination of clinical assessment, serum biomarkers, electroencephalography, somatosensory-evoked potentials, and neuroimaging is recommended during the early phase at 3–5 days after cardiac arrest. However, a poor outcome cannot be predicted with certainty and the assessment may be confounded by contradictory results from the different assessment modalities [4, 5]. Therefore, there is an unmet need for new biomarkers to improve the accuracy of early-phase prognostication in order to identify patients with a likely poor neurological outcome.

Among conventional methods of neuroimaging, the value of diffusion-weighted imaging (DWI) in neurological prognostication is well-demonstrated; however, DWI has been reported to underestimate the extent of ischemic injury during the first three days after OHCA [6]. While the gray matter has classically been thought to be more sensitive to hypoxic-ischemic brain damage, white matter is also highly vulnerable even in the early stages of ischemia [7]. Diffusion tensor imaging (DTI) is an extension of DWI that allows the evaluation of microstructural integrity of brain white matter using directional assessment of water diffusion, thus potentially being more sensitive than DWI to detect white matter damage in OHCA patients [8].

Proton magnetic resonance spectroscopy (1H-MRS) is another advanced magnetic resonance technique with some evidence of prognostic value in hypoxic-ischemic brain damage in adults after stroke and cardiac arrest as well as in asphyxiated neonates [9–11]. 1H-MRS can be used for detecting concentrations of several brain metabolites: *N*-acetylaspartate (NAA) is almost exclusively cited in neurons and is considered as a marker for neuronal integrity [12]. Creatine (Cr) is produced in the liver and is used as an energy source for ATP synthesis [13]. Often the sum signal of creatine and phosphocreatine (tCr) is used as a reference for other metabolites although its level does not remain constant in all pathologies [14]. Choline (Cho) and phosphocholine (PCho) are mainly situated in the cell membrane and considered as markers for cell membrane density and integrity [10].

However, the value of DTI and 1H-MRS, either alone or in combination with serum neuron-specific enolase (NSE), for predicting poor neurological outcomes at an early phase after OHCA, has yet to be established.

DTI performed between 7 and 28 days after cardiac arrest has shown great promise to accurately predict neurological outcome in this patient group [11]. As defined in the original study protocol, the purpose of this study was to test the hypothesis that DTI and/or MRS could be used at an earlier phase to assess the neurological outcome in this patient group and to further explore whether there is benefit in combining NSE with brain imaging. We assessed the predictive values for a 6-month neurological outcome, dichotomized as good (mRS 0–2) and poor (mRS 3–6), of fractional anisotropy from DTI, several brain metabolites from 1H-MRS each obtained by MRI (magnetic resonance imaging) and NSE performed in comatose survivors within 72 h after OHCA.

## Methods

### Study design

This study was approved by the ethics committee of the Hospital District of Southwest Finland and the institutional review boards of the Helsinki University Hospital and the Finnish Medicines Agency. All patients’ next of kin or legal representative gave written informed assent within 4 h after hospital arrival. Consent was sought from patients when they regained consciousness. An independent data and safety monitoring committee reviewed data after the enrolment of every 4 patients and after each 6-month interval. The study was conducted according to good clinical practice and the latest revision of the Declaration of Helsinki. The study design and methodology were consistent with the STARD guidelines for reporting diagnostic accuracy studies [15].

### Participants

Consecutive comatose survivors of witnessed out-of-hospital cardiac arrest from an initial shockable rhythm admitted to the Turku and Helsinki University hospitals between August 2009 and September 2014 were screened for eligibility. Detailed inclusion and exclusion criteria are listed in Supplementary Information (Online Resource 1).

### Randomization and blinding

The patients were allocated in a 1:1 ratio with random block sizes of 4, 6, and 8 to receive either temperature management with 33 °C (TTM) alone for 24 h or inhaled xenon LENOXe, Air Liquide Medical GmbH, DÜsseldorf, Germany in combination with TTM for 24 h. The neurological end-point evaluators as well as the patients were blinded to the treatment.

### Procedures

MRI imaging was scheduled to be performed within 16 h of rewarming, i.e., 36–52 h after OHCA. Patients were kept intubated and sedated (with sedation interruptions after completion of rewarming) until brain imaging was performed, regardless of neurological status. A predetermined prognostication protocol (see Supplementary Information Online Resource 1) was used to preclude premature decisions to withdraw life-sustaining therapy. DTI and 1H-MRS results did not inform the outcome of the prognostication. The clinical outcome was evaluated at 6 months after OHCA with a modified ranking scale (mRS) by experienced neurologists.

After rewarming was completed, sedation interruptions were initiated and performed every 6 to 12 h throughout the intensive care stay. A motor score of the Glasgow Coma Scale was assessed during each sedation interruption by a trained intensive care nurse or on-duty intensive care physicians. NSE serum concentration (Immuno-Electro-Chemi-Luminescent assay, Roche Diagnostics GmbH, Mannheim, Germany) was determined at hospital arrival, and at 24 h, 48 h, and 72 h after OHCA. An electroencephalogram was recorded only if it was clinically indicated.

Siemens Magnetom Verio 3 T scanner (Siemens Medical Solutions, Erlangen, Germany) with a 12-element Head Matrix coil was used in both MRI centers. DTI and DWI data were acquired using a diffusion-weighted spin-echo echo planar imaging (SE-EPI) sequence with 20 diffusion encoding directions (see Supplementary Table S1 in Online Resource 1 for details).

FSL software library (version 6.0, Analysis Group, FMRIB, Oxford, United Kingdom) was used for processing the DTI images, following the tract-based spatial statistics (TBSS) processing [16, 17]. This observer-independent and hypothesis-free method has the ability to spatially locate group differences in the DTI data. The mean fractional anisotropy value of white matter was calculated as a mean value of all the voxels in the skeleton (see Supplementary Methods in Online Resource 1 for details).

**Fig. 1:**
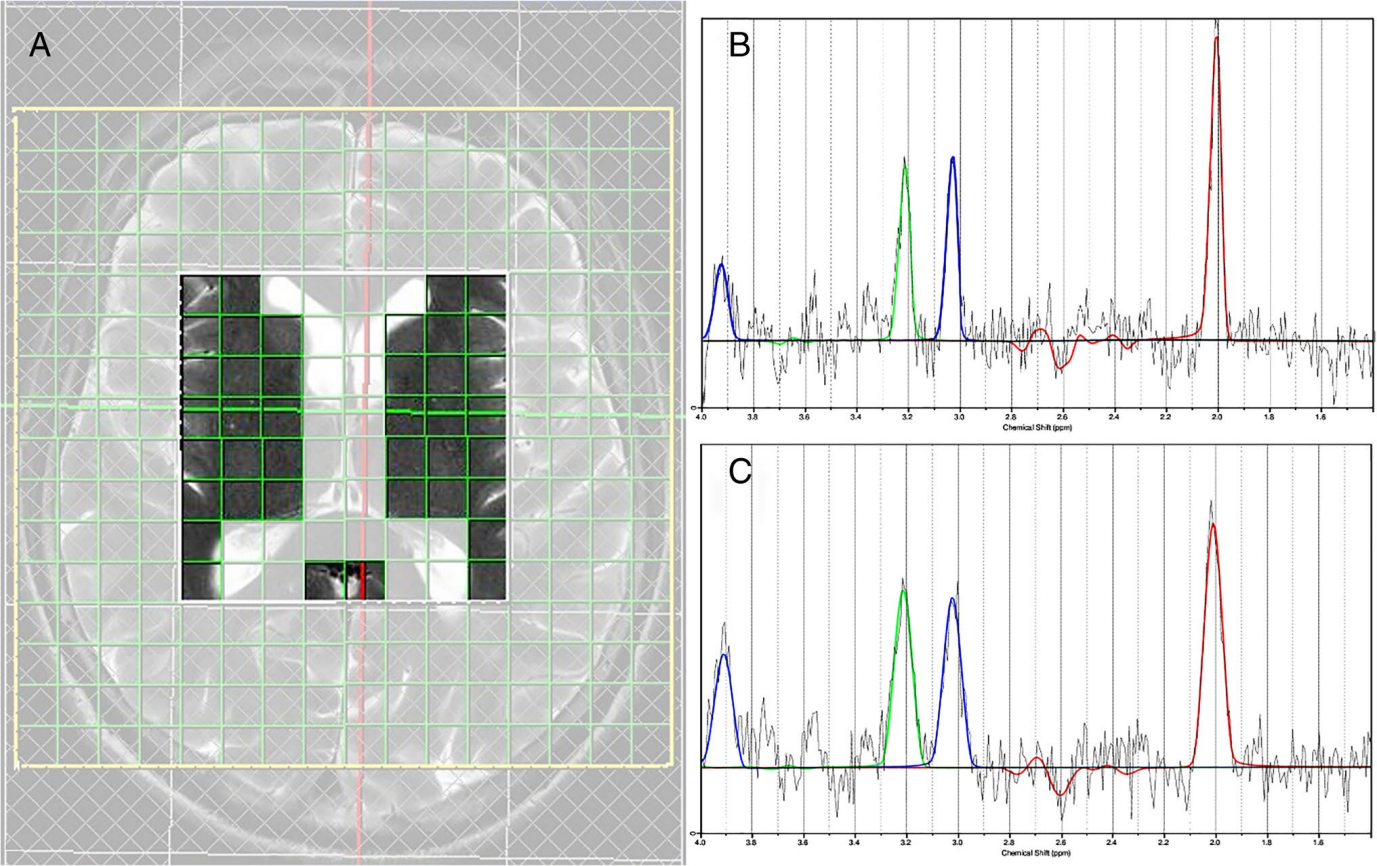
1H-MRS analyzed voxels and typical spectra. **A** An average of all analyzed voxels in basal ganglia excluding the ones typically including cerebrospinal fluid was used in the analysis. Voxels outside the analysis area are dimmed. **B** A typical spectrum of a single voxel with tNAA/tCr = 2.41 which is close to the mean of the survivors. **C** A typical spectrum of a single voxel with tNAA/tCr = 2.19 which is close to the mean of the non-survivors. The signal of total *N*-acetylaspartate is colored in red, total choline is colored in green, and total creatine is colored in blue

1H-MRS data were acquired from the region of basal ganglia by utilizing Chemical Shift Imaging (CSI) technique (see Supplementary Table S2 in Online Resource 1 for details). Acquired data were analyzed using the LCModel software (version 6.3-0C) [18]. An average of all analyzed voxels, except the ones containing cerebrospinal fluid (CSF), was selected for the final analysis (Fig. 1). The metabolite concentration values were corrected for relaxation effects (Supplementary Methods in Online Resource 1), but absolute concentration values were not feasible to use. Therefore, the amount of tNAA (total *N*-acetyl aspartate) and total choline (tCho) were expressed as ratios over total creatine (tCr), i.e., tNAA/tCr and tCho/tCr, as it is expected to remain stable. In addition, apart from the tNAA/tCho ratio, the ratio of tNAA and tCho was considered as these individual parameters are related to neuronal density, activity, and integrity [19].

### Statistical analysis

The sample size of 110 patients was based on a power analysis of the fractional anisotropy values from brain magnetic resonance imaging. The categorical demographic data and baseline clinical characteristics between groups of mRS 0–2 and mRS 3–6 were compared with chi-square or Fisher’s exact test. Two-sample *t* test or Mann–Whitney *U* test was used to test the differences in continuous demographic data and baseline clinical characteristics between the groups mRS 0–2 and mRS 3–6. The normality of continuous variables was evaluated visually using histograms. The mean differences in mean fractional anisotropy, 1H-MRS data, and NSE at 48 and 72 h after OHCA between the groups were tested with two-sample *t* test. Age-, sex-, treatment-, and site-adjusted mean differences between the groups were compared with analysis of covariance. NSE values were log-transformed for statistical analysis due to positively skewed distribution. Permutation-based voxel-wise statistical analysis with tract-based spatial statistics in conjunction with family-wise error correction was used for multiple comparisons across space to obtain group differences in the white matter tracts [16, 17].

The prognostic values of fractional anisotropy, tNAA/tCr, and NSE 72 h after OHCA and logistic regression-derived combined models were evaluated by calculating the area under the curve of the receiver operating characteristic curve (AUROC) using a nonparametric method. Sensitivity, specificity, positive predictive value (PPV), and negative predictive value (NPV) for each prognostic variable were calculated. Optimal cutoff values were chosen by using the Youden Index (sensitivity + specificity-1). Combined models were described as exploratory.

A 2-sided *p* value less than 0.05 was considered statistically significant. Statistical analyses were performed with SAS System for Windows, version 9.4 (SAS Institute Inc., Cary, NC) and SPSS Statistics for Macintosh, version 24 (IBM Corp., Armonk, NY).

## Results

### Patients

**Fig. 2:**
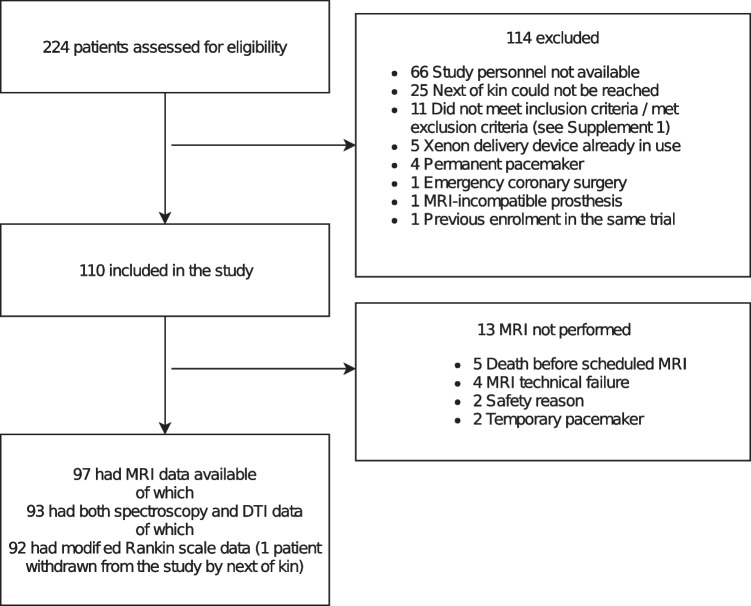
Flow of the participants

Of the 224 patients screened for eligibility, 110 were included. Of these, 97 underwent magnetic resonance imaging in a median (inter-quartile range) time of 53 h (47–64) after OHCA and 93 had 1H-MRS, DTI data available (Fig. 2).

One patient was withdrawn 6 days after the index event by the next of kin, and therefore, 92 out of 93 had applicable mRS data (Fig. 2, Supplementary Table S3 in Online Resource 1). Six months after OHCA, 61 patients had a good (mRS 0–2) and 31 patients had a poor (mRS 3–6) neurological functional outcome. Patient demographics and clinical characteristics are presented in Table 1.

**Table 1:** Demographic data and clinical characteristics of the patients

	All *n* = 93	mRS 0–2 *n* = 61	mRS 3–6 *n* = 31	*P* value
Baseline characteristics				
Age, years, median (IQR)	61.0 (54.5 − 67.0)	58.0 (53.0 − 64.0)	64.0 (57.0 − 71.0)	0.0029
Male sex, *n* (%)	69 (74.2)	43 (70.5)	25 (80.6)	0.2945
Coronary artery disease, *n* (%)	70 (75.3)	44 (72.1)	26 (83.9)	0.2121
Hypertension, *n* (%)	42 (45.2)	27 (44.3)	15 (48.4)	0.7073
Congestive heart failure, *n* (%)	7 (7.5)	4 (6.6)	3 (9.7)	0.6842
Diabetes, *n* (%)	13 (14.0)	6 (9.8)	7 (22.6)	0.1190
Asthma or chronic obstructive pulmonary disease, *n* (%)	13 (14.0)	9 (14.8)	4 (12.9)	1.000
Dyslipidemia, *n* (%)	35 (37.6)	20 (32.8)	14 (45.2)	0.2451
Smoker, *n* (%)	35 (38.9)^a^	21 (34.4)^b^	14 (45.2)	0.3763
Previous stroke, *n* (%)	21 (22.6)	11 (18.0)	20 (64.5)	0.1244
Selected laboratory values				
pH, median (IQR)	7.32 (7.29 − 7.37)^c^	7.33 (7.30 − 7.39)^e^	7.32 (7.27 − 7.35)^h^	0.1132
Lactate, µmol/l, median (IQR)	1.94 (1.30 − 2.80)^d^	1.80 (1.15 − 2.70)^f^	2.50 (1.55 − 3.24)^h^	0.0922
Creatine, µmol/l, median (IQR)	88.0 (77.0 − 111.8)^c^	85.0 (74.0 − 103.0)^g^	101.5 (83.0 − 145.0)^i^	0.0046
Resuscitation details				
Bystander resuscitation, n (%)	67 (72.0)	46 (75.4)	20 (64.5)	0.2727
Delay in EMS, min, mean (SD)	8.6 (3.4)	8.7 (3.1)	8.2 (4.1)	0.5041
ROSC, min, mean (SD)	22.0 (6.8)	20.2 (6.3)	25.3 (6.7)	0.0005
No flow, min, median (IQR)	0.0 (0.0 − 4.0)	0.0 (0.0 − 0.0)	0.0 (0.0 − 6.0)	0.2546
Cooling procedure details				
Core temperature before the start of cooling, °C, mean (SD)	35.04 (1.25)	35.13 (1.26)	34.8 (1.26)	0.3088
Time from OHCA to target temperature, min, median (IQR)	311 (263 − 370)	295 (246 − 354)	354 (291 − 406)	0.0194
Cooling rate, °C /h, median (IQR)	0.42 (0.25 − 0.50)	0.43 (0.29 − 0.56)	0.36 (0.14 − 0.47)	0.263
Clinical characteristics during ICU stay				
ES, *n* (%)	26 (28.0)	6 (9.8)	19 (61.3)	< 0.0001
STEMI, *n* (%)	34 (36.6)	19 (31.1)	15 (48.4)	0.1054
NSTEMI, *n* (%)	54 (58.1)	37 (60.7)	16 (51.6)	0.4068
Acute kidney injury, *n* (%)	20 (21.5)	12 (19.7)	8 (25.8)	0.5001
Xenon group, *n* (%)	47 (50.5)	31 (50.8)	15 (48.4)	0.8254
Norepinehrine cumulative dose of 72 h after ICU admission, mg, median (IQR)	16.5 (6.8 − 33.8)	13.5 (6.0 − 30.2)	30.4 (17.9 − 45.1)	0.0017

Data are expressed as numbers (percentage) unless otherwise indicated

Due to missing data, mRS was applicable for *N* = 92

Due to missing data, smoking is applicable for ^a^*N* = 90 and ^b^*N* = 59

Due to missing data, laboratory values are applicable for ^c^*N* = 79, ^d^*N* = 78, ^e^*N* = 55, ^f^*N* = 54, ^g^*N* = 56, ^h^*N* = 24, and ^i^*N* = 23

*mRS* modified ranking scale, *IQR* interquartile range, *EMS* emergency medical service, *ROSC* return of spontaneous circulation, *ES* Epileptic seizures, *STEMI* ST-elevation myocardial infarction, *NSTEMI* non-ST-elevation myocardial infarction

### NSE, motor score, and epileptic seizures

NSE at 48 and 72 h after OHCA was significantly higher in patients with poor neurological outcomes than in the patients with good neurological outcomes at 6 months (Table 2, Supplementary Table S4 in Online Resource 1). Nine of the 61 patients with good neurological outcomes and 27 out of 31 patients with poor neurological outcomes had motor score ≤ 3 at 72 h after OHCA (Table 2). Ten patients responded appropriately to commands within 48 h after OHCA; a further 32 patients were responsive to commands between 48 and 72 h, and 25 patients achieved this state later than 72 h. Twenty-five patients never achieved a motor score of 6, all of whom died. The proportion of patients with epileptic seizures (ES) was also significantly higher in the poor outcome group (19/31) than in the good outcome group (6/61).

**Table 2:** Prognostic variables in patients with good and poor neurological outcome at six months after OHCA

	Unadjusted Mean (SD)		Mean difference (95% CI)		*P* value	
	mRS 0–2 (*n* = 61)	mRS 3–6 (*n* = 31)	Unadjusted	Adjusted ^a^	Unadjusted	Adjusted ^a^
tNAA/tCr	2.43 (0.21)	2.18 (0.22)	0.25 (0.15 to 0.34)	0.22 (− 0.12 to 0.31)	< 0.0001	< 0.0001
tCho/tCr	0.35 (0.04)	0.35 (0.04)	0.01 (− 0.01 to 0.03)	0.01 (− 0.01 to 0.02)	0.3831	0.5403
tNAA/tCho	6.96 (0.91)	6.37 (0.68)	0.59 (0.22 to 0.96)	0.54 (0.16 to 0.93)	0.0020	0.0065
Fractional anisotropy	0.43 (0.02)	0.41 (0.03)	0.03 (0.01 to 0.04)	0.02 (0.01 to 0.03)	< 0.0001	0.0014
Basal ganglia MD, 10^–6^ mm^2^/s	807.3 (54.8)	809.8 (87.9)	− 2.53 (− 32.18 to 27.13)	3.20 (− 27.92 to 34.31)	0.8659	0.8387
NSE 48 h^b^	2.97 (0.38)	3.71 (0.71)	− 0.73 (− 0.96 to − 0.51)	− 0.74 (− 0.98 to − 0.49)	< 0.0001	< 0.0001
NSE 72 h^b,c^	2.71 (0.48)	3.65 (0.87)	− 0.94 (− 1.22 to − 0.66)	− 0.91 (− 1.21 to − 0.61)	< 0.0001	< 0.0001
			Unadjusted odds ratio (95 CI)	Adjusted ^a^ odds ratio (95 CI)		
GCS motor scores 1–3 at 72 h, *n*(%)	9 (14.8)	27 (87.1)	38.99 (10.9 to 138.34)	37.31 (9.85 to 141.32)	< 0.0001	< 0.0001
ES, *n*(5)	6 (9.8)	19 (61.3)	14.51 (4.78 to 44.05)	19.93 (5.27 to 75.44)	< 0.0001	< 0.0001

^a^Data are adjusted for age, sex, site, and treatment group

^b^Values were log-transformed for statistical analysis

^c^Due to missing data NSE 72 h values are applicable for *n* = 60 in mRS 0–2 patients

Values are mean (SD) unless otherwise indicated

*mRS* modified ranking scale, *CI* confidence intervals, *tNAA* total *N*-acetyl aspartate, *tCho* total choline, *tCr* total creatine, *NSE* neuron-specific enolase, *GCS* Glasgow Coma Scale, *ES* epileptic seizures

### DTI and 1H-MRS results

**Fig. 3:**
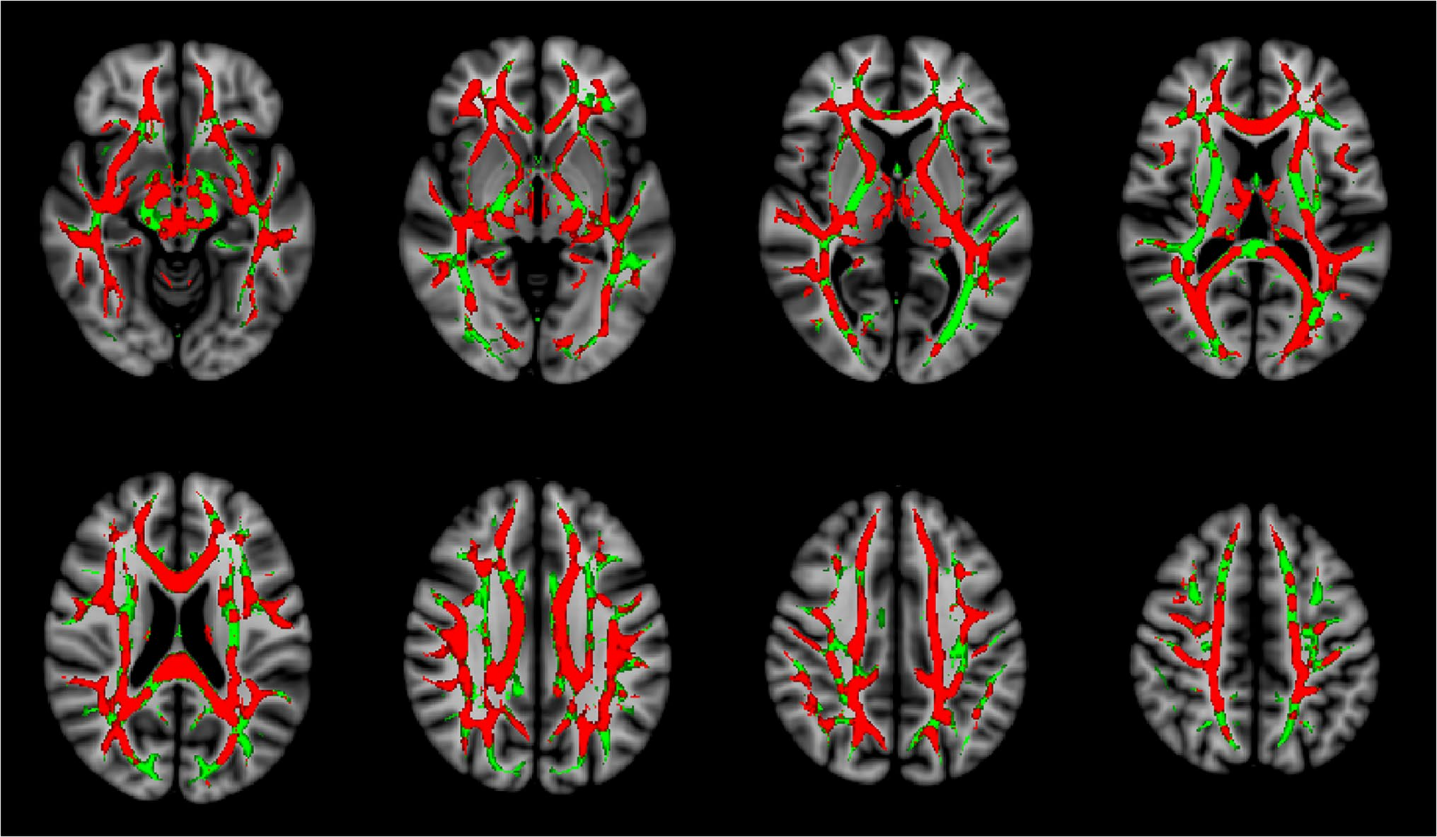
White matter damage leads to a loss of microstructural organization that can be quantified by the loss of directionality in the diffusion of water molecules in the white matter tracts. Fractional anisotropy is a scalar value representing this directionality of water diffusion; lower fractional anisotropy values are indicative of less organized diffusion and are an index of more extensive white matter damage. The visualization presents the results of the voxel-wise tract-based spatial statistics analysis of fractional anisotropy values between patients with good (mRS 0–2) and poor (mRS 3–6) 6-month neurological outcomes. Voxels with significantly (*P* < 0.05, family-wise error corrected for multiple comparisons) lower fractional anisotropy values in patients with poor neurological outcome were identified and are shown in red in the statistical visualization (i.e., 57.9% of all 123,994 analyzed voxels), whereas the areas in which there was no significant difference in fractional anisotropy values between the groups are shown in green (i.e., 42.1% of all analyzed voxels). According to the Johns Hopkins University white matter tractography atlas [20], the tract-wise distribution of the voxels (percentages in parentheses below) with significantly (*P* < 0.05; family-wise error corrected for multiple comparisons) lower fractional anisotropy in non-survivors (marked red in the figure) were as follows: cingulum (cingulate gyrus) (41.6%), cingulum (hippocampal region) (59.6%), forceps minor (74.9%) and major (51.6%), superior longitudinal fasciculus (61.6%), inferior longitudinal fasciculus (57.3%), anterior thalamic radiation (66.0%), inferior fronto-occipital fasciculus (62.5%), corticospinal tract (56.4%), uncinate fasciculus (72.4%), and the body of corpus callosum (87.4%)

Mean fractional anisotropy values of the DTI, and tNAA/tCr and tNAA/tCho ratios of the 1H-MRS were significantly higher in patients with mRS 0–2 than in patients with mRS 3–6 (Table 2). The result of the tract-based spatial statistics analysis is visualized with a statistical parametric map (Fig. 3) [20].

### Receiver operating characteristic analysis of the single measures

**Fig. 4:**
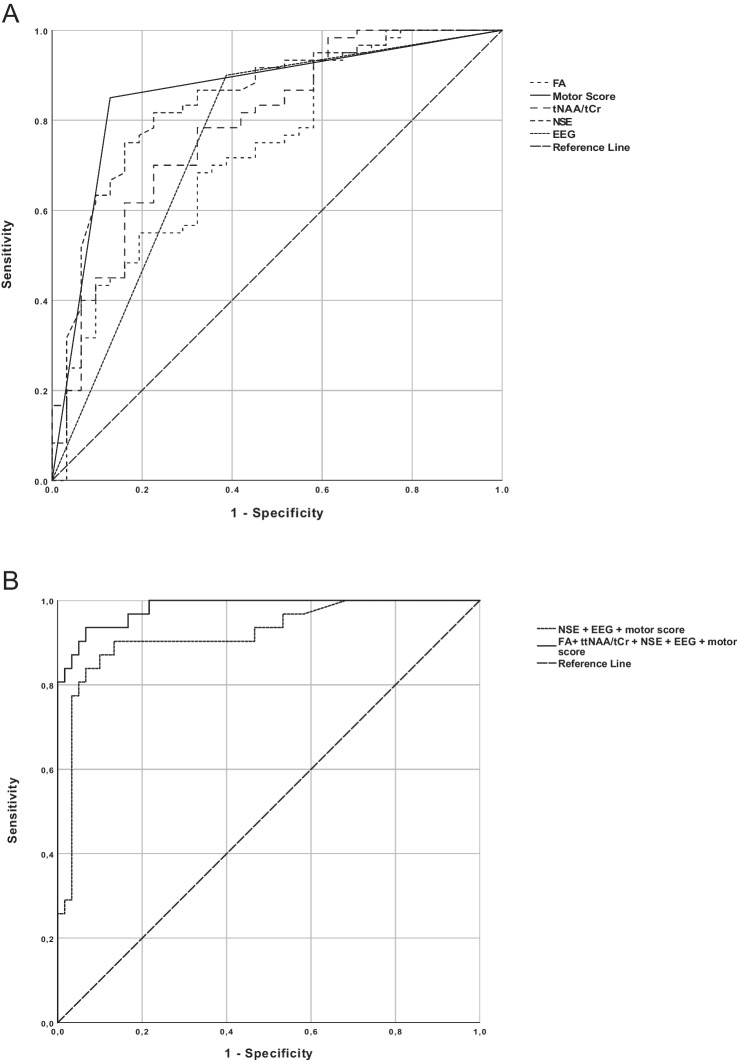
Receiver operating characteristic curves of single measures of fractional anisotropy, tNAA/tCr, EEG (epileptic seizures), and NSE at 72 h and motor score ≤ 3 at 72 h (**A**). The combination of EEG (epileptic seizures), motor score, and NSE at 72 h, and the combination of fractional anisotropy, tNAA/tCr, EEG (epileptic seizures), NSE and motor score ≤ 3 at 72 h (**B**)

The AUROC for predicting poor outcome was 0.73 (95% CI 0.62–0.84) for fractional anisotropy of DTI, 0.78 (0.68–0.88) for tNAA/tCr of 1H-MRS, and 0.85 (0.76–0.93) for NSE at 72 h; fractional anisotropy vs tNAA/tCr, fractional anisotropy vs NSE, and tNAA/tCr vs NSE; *p* = 0.53, *p* = 0.13, and *p* = 0.38, respectively (Fig. 4A, Table 3). Motor score at 72 h provided the best diagnostic predictive value for poor neurological outcome (AUROC 0.88 (95% CI 0.80–0.96), but it did not differ significantly with AUROC values of fractional anisotropy (*p* = 0.058), tNAA/tCr (*p* = 0.16), or NSE at 72 h (*p* = 0.51). The predictive value for ES (AUROC 0.76 (0.66–0.85)) was comparable to neuroimaging (Table 3, Fig. 4A).

**Table 3:** Area under the receiver operating characteristic curve (AUROC) values for prognostic variables and their combinations in patients with a poor neurological outcome at 6 months after OHCA

	AUROC	95% CI	Cutoff	Sensitivity	Specificity	Positive predictive value	Negative predictive value
tNAA/tCr	0.78	0.68 to 0.88	≤ 2.313	0.77	0.69	0.56	0.86
tCho/tCr	0.58	0.45 to 0.71	≤ 0.3445	0.61	0.59	0.43	0.75
tNAA/tCho	0.69	0.58 to 0.80	≤ 7.154	0.97	0.39	0.45	0.96
Fractional anisotropy	0.73	0.62 to 0.84	≤ 0.4235	0.68	0.69	0.53	0.81
NSE 48 h	0.84	0.74 to 0.94	≥ 25	0.84	0.79	0.67	0.91
NSE 72 ha	0.85	0.76 to 0.93	≥ 21	0.77	0.82	0.69	0.88
GCS motor score at 72 h	0.88	0.80 to 0.96	≤ 3	0.87	0.85	0.75	0.93
ES	0.76	0.66 to 0.85	yes	0.61	0.90	0.76	0.82
tNAA/tCr + Fractional anisotropy	0.83	0.75 to 0.91	NA	0.90	0.59	0.53	0.92
GCS motor score at 72 h ≤ 3 + NSE 72 h + ES	0.92	0.85 to 0.98	NA	0.84	0.93	0.87	0.92
GCS motor score at 72 h ≤ 3 + NSE 72 h + ES + tNAA/tCr + Fractional anisotropy	0.98	0.96 to 1.00	NA	0.94	0.93	0.88	0.97
^a^Due to missing data, NSE 72 h values are applicable for *n* = 60 in mRS 0–2 patients							

*AUROC* the area under the receiver operating characteristic curve, *CI* confidence intervals, *tNAA* total *N*-acetyl aspartate, *tCho* total choline, *tCr* total creatine, *NSE* neuron-specific enolase, *GCS* Glasgow Coma Scale *mRS* modified ranking scale, *ES* epileptic seizures

### Explorative analysis

The combination of fractional anisotropy, tNAA/tCr, ES, and motor score at 72 h improved the diagnostic performance with or without NSE at 72 h (AUROC 0.98, 95% CI 0.96–1.00) as compared with NSE, motor score, and ES without imaging studies (AUROC 0.92 (0.85–0.98), *p* = 0.037) (Table 3, Fig. 4B).

### Subgroup analysis

A subgroup of 46 patients had a motor score below 6 at 72 h after OHCA. The AUROC for predicting poor outcome in this subgroup was just slightly smaller than in the whole data: 0.72 (95% CI 0.57–0.87) for DTI (fractional anisotropy), 0.76 (0.62–0.90) for 1H-MRS (tNAA/tCr), and 0.82 (0.69–0.95) for NSE at 72 h; fractional anisotropy vs tNAA/tCr, fractional anisotropy vs NSE, and tNAA/tCr vs NSE; *p* = 0.65, *p* = 0.33 and *p* = 0.55, respectively.

## Discussion

The main finding of this study was that early-stage quantitative fractional anisotropy of DTI or 1H-MRS did not perform better than NSE or motor score at 72 h or ES for prognosticating poor outcomes at 6 months after OHCA. Combining fractional anisotropy and tNAA/tCr to NSE, motor score and ES improved diagnostic accuracy slightly.

Of the single measures, motor score of ≤ 3 at 72 h provided the best diagnostic predictive value for poor neurological outcomes and NSE at 72 h was the next best. However, the predictive value of the motor score did not reach statistical significance as compared with the results revealed by NSE at 72 h or by the quantitative neuroimaging. In earlier studies, a cutoff value ≤ 2 for motor score has revealed low specificity and high sensitivity between 70 and 80% [21, 22]. Here, we demonstrate similar sensitivity values with higher specificity with the cutoff value of ≤ 3, which is consistent with the latest guidelines [4]; a homogenous cardiac arrest population that only included patients with a shockable primary rhythm can also explain the improvement in the latter attribute.

The current AUROC of 0.85 for NSE is consistent with the values of 0.86 and 0.90 recorded in earlier large studies in TTM-treated patients [23, 24]. However, comparing NSE results among studies may be problematic because cutoff values vary and a consistent threshold limit for 0% false positive ratio has not been recommended [4]. However, the recent guidelines identified a cutoff value of over 60 µg/L at 72 h to suggest poor prognosis when used as part of the multimodal prognostication [4]. In this study, the predictive value of NSE at 72 h with a cutoff value of 21 as identified by the Youden Index did not provide significantly better diagnostic performance as compared to motor score or single parameters obtained by the brain MRI.

Fractional anisotropy is a DTI-derived scalar value that reflects white matter tissue characteristics such as a fiber density, organization coherence, myelination, and axon diameter [25]. Lower fractional anisotropy values in ischemic white matter probably represent a combination of myelin damage, axonal degeneration, and edema, which all contribute to the loss of directional diffusion in white matter tracts [26].

A very recent study demonstrated a prognostic value of decreased mean global white matter fractional anisotropy levels imaged 7 to 28 days after cardiac arrest for long-term neurological outcome with an AUROC of 0.95 in a subset of 150 patients with a persistent unresponsiveness at day 7 [11]. According to earlier evidence, most survivors regain consciousness within a week and usually all of them within 10 days after cardiac arrest [4, 5, 27–29]. However, Velly and colleagues revealed that as many as 22% of patients who were without a response to simple commands a week after cardiac arrest may still have a favorable outcome 6 months after cardiac arrest [11]. The current lower prognostic value for fractional anisotropy at the early subacute phase can be partly due to evolving white matter injury over time. This suggests that the predictive value of fractional anisotropy varies depending on the used imaging time window [30]. Conventional DWI MRI seems to perform better than DTI/1H-MRS in the early phase [31, 32]. However, in this study, conventional DWI did not provide any additional value.

NAA is ubiquitous in the central nervous system and is a marker of the integrity of mature neurons; persistent reductions in NAA have been used as a marker of neuronal loss and extent of neurological damage and have prognostic value for outcome in stroke and cardiac arrest [9, 11, 33]. These earlier interpretations were supported by the present study revealing that ratios of tNAA/tCr and tNAA/tCho in the basal ganglia are independent predictors for poor neurological outcomes at 6 months.

In explorative analysis, the addition of early fractional anisotropy of DTI and tNAA/tCr to ES, NSE, and motor score at 72 h provided the best diagnostic performance with an AUROC of 0.98 with or without NSE. This was significantly better than the next best model including ES, motor score at 72 h and NSE. However, the absolute difference in diagnostic value remained relatively small and the predictive value without neuroimaging was already very good with an AUROC of 0.92. Therefore, considering the cost and challenges of MR imaging of critically-ill patients, early-phase DTI/MRS cannot be recommended for routine clinical practice, although it may have a potential role as an additional imaging modality in a multimodal prognostication for selected cases. The clinical applicability of DTI/MRS in the early phase after cardiac arrest needs further investigations in another population including patients with shockable and non-shockable primary rhythms.

There are some limitations in this study. First, our results represent a two-center (single country) cohort of patients in successfully resuscitated cardiac arrest victims in whom a shockable rhythm was the initial rhythm at the time of resuscitation. Therefore, further validation of the results is required in patients with asystole and pulseless electrical activity. Second, earlier studies have demonstrated that pupillary reactivity assessed with pupillometry has a high accuracy to predict poor outcomes after cardiac arrest [34]. Unfortunately, a predictive value of pupillary light reflexes could not be analyzed due to methodological reasons. In this study, the pupillary reactivity was assessed only by standard visual inspection, which led to missing values in 61 patients. Third, metabolite concentration ratios instead of absolute values or inter-subject metabolite concentrations were used. The use of absolute concentration values would have required the use of reference solutions for calibrations and information about coil loading and T2 attenuation, or the use of a water-referencing method; none of these options was available for this study. In routine clinical practice, absolute metabolite values are seldom available, and thus, this approach was not deemed feasible. Fourth, lactate would be an important metabolite in evaluating the extent and stage of the neuronal damage. Unfortunately, the echo time of 135 ms in the CSI sequence leads to an unreliable determination of lactate signal in the 3 T MRI scanner. For a more robust detection of lactate, an echo time of 288 ms should be used, although it would decrease the overall SNR of the spectra [35]. Fifth, due to a possible bias caused by the neuroprotective effect of the xenon on the prognostic value of the current metrics, all statistical analyses were adjusted with the treatment group. Finally, our model was limited by a small number of patients with the study endpoint, and therefore, all variables with predictive value for poor outcomes could not be included.

## Conclusions

Neither early-stage quantitative fractional anisotropy measured by DTI nor tNAA/tCr measured by 1H-MRS was better than NSE or motor score at 72 h in predicting poor outcomes in this patient group. While early-phase quantitative neuroimaging provided a statistically significant improvement in the predictive value when combined with NSE, motor score, and ES, the effect was small. Therefore, early-phase DTI/MRS cannot be recommended for routine clinical practice, although it may have a role in multimodal prognostication in specific cases. Current results warrant validation in a larger prospective study in cardiac arrest patients with shockable and non-shockable primary rhythms.

## Supplementary Information

Below is the link to the electronic supplementary material.

Supplementary file1 (pdf 51.8 KB)

## Data Availability

The datasets used and/or analyzed during the current study are available from the corresponding author on reasonable request.
