# Supplementary material for: Comparison of the prognostic value of early-phase proton magnetic resonance spectroscopy and diffusion tensor imaging with serum neuron-specific enolase at 72 h in comatose survivors of out-of-hospital cardiac arrest—a substudy of the XeHypotheca trial
Source: Neuroradiology. 2022 Oct 17;65(2):349–60. doi: 10.1007/s00234-022-03063-z (PMC9859870; doi:10.1007/s00234-022-03063-z)
Supplement: Supplementary file 1 — Supplementary file1 (pdf 51.8 KB) [file 234_2022_3063_MOESM1_ESM.pdf]

Supplementary Information – Online resource 1

Table of contents

Neurological prognostication after out-of-hospital cardiac arrest in hypothermia treated patients (page 2)

Inclusion and exclusion criteria (page 3)

Supplementary Methods. Complete magnetic resonance imaging and spectroscopy protocol (page 4)

Supplementary Table S1. Magnetic resonance imaging parameters (page 5)

Supplementary Table S2. Proton magnetic resonance spectroscopy parameters (page 6)

Supplementary Table S3. Neurological outcome at six months after out-of-hospital cardiac arrest in the study group using the modified Rankin Scale score (page 7)

Supplementary Table S4. Results of NSE in patients with good and poor neurological outcome at 6-months (page 8)

References of the Supplementary Information (page 9)

This supplementary material has been provided by the authors to give readers additional information about their work.

Neurological prognostication after out-of-hospital cardiac arrest in hypothermia treated patients

Neurological prognostication is accomplished if the patient remains unconscious 12 h after rewarming. All sedative medication must have been discontinued 12 h previously, and in hypothermia treated patients prognostication is performed after 72 h post resuscitation.
Neurological examination is performed by consultant neurologist consisting:

1. Glasgow Coma Scale (eye, speech and movement responses)
2. Brain stem reflexes
3. Breathing
4. Possible myoclonus

Assessments:
If the patient remains unconscious:

1. Brain CT or MRI scan is performed
2. EEG is obtained to exclude convulsive or nonconvulsive status epilepticus (NCSE). The key considerations:
   Continuity of electrical activity of the brain
   Response to external stimuli
   Spontaneous dynamics in brain electrical activity
   Presence of epileptiformic waves
   Non-convulsive status epilepticus

Serum neuron specific enolase (NSE) is assessed at 24 h and 48 h (±2 h) after OHCA

Sensory evoked potential (SEP) assessment is performed if CT/MRI and EEG provide no explanation to unconsciousness

Conclusion
As signs for poor prognosis are:

1. CT/MRI: general cerebral edema with sulcal effacement and wide-spread ischaemia with loss of margins of brain white and grey matter
2. EEG: generalized suppression (<20 μV) or burst suppression, generalized epileptic activity or periodic epileptiformic discharges (PED) with background activity suppression, lack of spontaneous variation and lack of reactivity to external stimuli
3. Continuous refractory to treatments myoclonic status epilepticus with permanent unconsciousness
4. Serum NSE values: ascending trend 24-48 h
5. Unresponsiveness to painful stimuli or extension as the best motor response at 72 h
6. Absent brain stem reflexes at 72 h
7. Bilateral absence of thalamocortical sensory evoked potentials (SEP)
8. Generalized diminished cortical diffusion on MRI

Inclusion and exclusion criteria

Inclusion criteria

- Witnessed cardiac arrest
- Ventricular fibrillation
- Non-perfusing ventricular tachycardia
- Presumed cardiac origin
- Age 18-80 years
- Start of resuscitation by emergency medical personnel within 15 minutes
- Return of spontaneous circulation within 45 minutes
- Decision for therapeutic hypothermia treatment by attending physician

Exclusion criteria

- Hypothermia (core temperature < 30°C)
- Unconsciousness before collapse (cerebral trauma, intoxication etc.)
- Computer tomography scan indicating cerebral pathological reason for cardiac arrest
- Responding to verbal commands after return of spontaneous circulation (ROSC)
- Pregnancy
- Coagulopathy
- Systolic arterial pressure less than 80mmHg lasting >30 minutes after ROSC
- Mean arterial pressure less than 60mmHg lasting <30 minutes after ROSC
- Hypoxemia (arterial oxygen saturation <85%) lasting >15 minutes after ROSC
- Factors making participation in follow-up implausible
- Enrolment in another interventional trial

Supplementary Methods. Complete magnetic resonance imaging and spectroscopy protocol

DTI image processing details

Preprocessing of the diffusion tensor imaging data was done using the DTIprep quality control software.[1] The following steps were performed: Diffusion information checks (ensuring correct diffusion gradient orientations, gradient b-values). Inter-slice brightness artifact detection via normalized correlation analysis between successive slices within a single DWI volume. Interlaced correlation analysis for detection and removal of "venetian blind" artifacts and motion within a single DWI volume. Co-registration to an iterative average over all the baseline images. Eddy-current and motion artifact correction, including appropriate gradient direction adjustments. Residual motion detection to ensure all DWI volumes are well registered.

The diffusion tensor image model was fitted using the dtifit tool (FSL 6.0, Analysis Group, FMRIB, Oxford, United Kingdom). FA images were aligned to a typical FA image in the dataset using the nonlinear registration tool FNIRT, which uses a b-spline representation of the registration warp field. Then aligned dataset was affine-transformed into 1x1x1mm3 standard space (MNI152). Following the default tract-based spatial statistics (TBSS) pipeline, all individual patients' spatially normalized fractional anisotropy images were projected onto a skeletonized mean fractional anisotropy map for statistical voxel-wise regression analysis between the two patient groups [2, 3]. The skeleton represented the centers of all white matter structures that were generally common to the subject involved in a study. Each subject's aligned, non-skeletonized FA data were then projected on the mean FA skeleton in such a way that each skeleton voxel takes the FA value from the local center of the nearest relevant tract.[3] These projected FA values were used for voxel wise statistical analysis.[2] Mean FA value of white matter was calculated as a mean value of all the voxels in the skeleton.

As described previously, the tract-wise distribution of percentages of voxels with significantly lower fractional anisotropy was also analysed.[4] Localization and labeling of the tracts were confirmed and identified with JHU white-matter tractography atlas.[5]

Metabolite exclusion criteria

Metabolite data with poor quality was omitted by using spectral and fitting quality parameters as exclusion criteria. The criteria were determined as follows: SNR had to be higher than 2 and FWHM lower than 0.15 ppm. Standard deviation of the fitting of tCr signal had to be less than 20%. Similar or corresponding limits have been used previously [6-8], however, in this study the fitting quality of tNAA was not included into the criteria to avoid affecting the correlations between the metabolite level and patient outcome, for example, level of tNAA might decrease close to zero in case of severe neuronal damage, thus making the fitting procedure of tNAA more prone to errors.

Corrections for T2 and T1 relaxation effects in 1H-MRS data

Metabolite concentration values (S_{measured}) were corrected for T2 and T1 relaxation effects as in [9] using the relaxation rates determined by Zaaraoui et al [10] as follows:

$$S_{\text{corrected}} = \frac{S_{\text{measured}}}{\exp(\frac{-TE}{T2})\left[1 - \exp(\frac{-TR}{T1})\right]}$$

where $S_{\text{corrected}}$ is the T2 and T1 corrected metabolite signal.

Supplementary Table S1. Magnetic resonance imaging parameters. FLAIR: Fluid Attenuated Inversion Recovery MP-RAGE: Magnetization Prepared Rapid Gradient Echo

MRI sequence	In-plane resolution (mm)	Slice thickness (mm)	Echo time (ms)	Repetition time (ms)
T2-weighted	0.4 x 0.4	4.0	96	5210
3D FLAIR	1.0 x 1.0	1.0	395	5000
T1-weighted (3D MP-RAGE)	1.0 x 1.0	1.0	2.2	1900
Diffusion tensor imaging[a]	2.0 x 2.0	3.0	100	6100

[a]Number of diffusion directions: 20, b-value 1000 s/mm^2, number of averages 2.

Supplementary Table S2. Proton magnetic resonance spectroscopy parameters

Volume selection method	Chemical Shift Imaging (CSI)
Matrix size	16 x 16 voxels
Volume of interest	8 x 8 voxels
Individual voxel size (left-right, anterior-posterior, head-feet)	10 mm x 10 mm x 15 mm
Sequence type	Point Resolved Spectroscopy Sequence (PRESS)
Echo time	135 ms
Repetition time	1700 ms
Spectral width	2000 Hz
Number of data points	1024
Number of signal averages	3
Water suppression method	Chemical-Shift-Selective (CHESS)
Shimming method	Automated

Supplementary Table S3. Neurological outcome at six months after out-of-hospital cardiac arrest in the study group using the modified Rankin Scale score

	Description of Category	All n = 92[a]
0	No symptoms	39 (42.4)
1	No significant disability: able to carry out all usual activities, despite some symptoms	11 (12.0)
2	Slight disability: able to look after own affairs without assistance, but unable to carry out all previous activities	11 (12.0)
3	Moderate disability: requires some help, but able to walk unassisted	2 (2.2)
4	Moderately severe disability: unable to attend to own bodily needs without assistance	2 (2.2)
5	Severe disability: requires constant nursing care and attention	0 (0.0)
6	Death	27 (29.4)

[a] One patient was withdrawn from the study 6 days after the index event

Supplementary Table S4. Results of NSE in patients with good and poor neurological outcome at 6-months (Mean (SD)).

	NSE 0 h	NSE 24 h	NSE 48 h	NSE 72 h
mRS(0-2)	21.6 (6.3)	24.2 (8.6)	21.2 (9.6)	17.3 (11.4)
mRS(3-6)	25.9 (10.8)	36.7 (20.5)	55.2 (61.7)	59.8 (78.9)

References of the Supplementary Information

1. Oguz I, Farzinfar M, Matsui J, Budin F, Liu Z, Gerig G, et al. DTIPrep: quality control of diffusion-weighted images. Front Neuroinform. 2014;8:4.

2. Smith SM, Jenkinson M, Johansen-Berg H, Rueckert D, Nichols TE, Mackay CE, et al. Tract-based spatial statistics: Voxelwise analysis of multi-subject diffusion data. Neuroimage. 2006;31(4):1487-505.

3. Jenkinson M, Beckmann CF, Behrens TE, Woolrich MW, Smith SM. Fsl. Neuroimage. 2012;62(2):782-90.

4. Laitio R, Hynninen M, Arola O, Virtanen S, Parkkola R, Saunavaara J, et al. Effect of Inhaled Xenon on Cerebral White Matter Damage in Comatose Survivors of Out-of-Hospital Cardiac Arrest A Randomized Clinical Trial. JAMA. 2016;315(11):1120-8.

5. Wakana S, Jiang H, Nagae-Poetscher LM, van Zijl PC, Mori S. Fiber tract-based atlas of human white matter anatomy. Radiology. 2004;230(1):77-87.

6. Doelken MT, Mennecke A, Stadlbauer A, Kecskeméti L, Kasper BS, Struffert T, et al. Multi-voxel magnetic resonance spectroscopy at 3 T in patients with idiopathic generalised epilepsy. Seizure. 2010;19(8):485-92.

7. Srinivasan R, Sailasuta N, Hurd R, Nelson S, Pelletier D. Evidence of elevated glutamate in multiple sclerosis using magnetic resonance spectroscopy at 3 T. Brain. 2005;128(Pt 5):1016-25.

8. Wilson M, Cummins CL, Macpherson L, Sun Y, Natarajan K, Grundy RG, et al. Magnetic resonance spectroscopy metabolite profiles predict survival in paediatric brain tumours. Eur J Cancer. 2013;49(2):457-64.

9. Soher BJ, van Zijl PC, Duyn JH, Barker PB. Quantitative proton MR spectroscopic imaging of the human brain. Magn Reson Med. 1996;35(3):356-63.

10. Zaaraoui W, Fleysher L, Fleysher R, Liu S, Soher BJ, Gonen O. Human brain-structure resolved T-2 relaxation times of proton metabolites at 3 Tesla. Magn Reson Med. 2007;57(6):983-9.
